# Reviewer acknowledgement 2014

**DOI:** 10.1186/s13013-015-0034-y

**Published:** 2015-02-22

**Authors:** Theodoros B Grivas

**Affiliations:** Orthopaedic & Traumatology Department, “Tzaneio” General Hospital of Piraeus, Piraeus, Greece

## Abstract

The Editor of *Scoliosis* would like to thank all our reviewers who have contributed to the journal in Volume 9 (2014).

**Emre Acaroglu**

Turkey

**Tamir Ailon**

USA

**Angelo Gabriele Aulisa**

Italy

**Lorenzo Aulisa**

Italy

**Marie Beauséjour**

Canada

**Viola Bullmann**

Germany

**Richard Burwell**

United Kingdom

**Federico Canavese**

France

**Rene Marten Castelein**

The Netherlands

**Jack Cheng**

Hong Kong

**Woojin Cho**

USA

**Nachiappan Chockalingam**

United Kingdom

**Winnie Chu**

China

**Alvin Crawford**

USA

**Peter Dangerfield**

United Kingdom

**Marinus de Kleuver**

The Netherlands

**Jean Claude de Mauroy**

France

**Sabrina Donzelli**

Italy

**Jean-Pierre Farcy**

USA

**John Ferguson**

New Zealand

**Thomas Findley**

USA

**Naglaa Gadallah**

Egypt

**Wojciech Glinkowski**

Poland

**Manabu Ito**

Japan

**Sasan Karimi**

USA

**Patrick Knott**

USA

**Tomasz Kotwicki**

Poland

**Toru Maruyama**

Japan

**Hitesh Modi**

India

**Alan Moulton**

United Kingdom

**Thomas Niemeyer**

Germany

**Hilali Noordeen**

United Kingdom

**David Nuckley**

USA

**James Ogilvie**

USA

**Joshua Pahys**

USA

**Eric Parent**

Canada

**Petros Patias**

Greece

**Gerald Quan**

Australia

**Dietrich Schlernzka**

Finland

**Wun-Jer Shen**

Taiwan

**Anuj Singla**

USA

**Paul Sponseller**

USA

**Luke Stikeleather**

USA

**Ian Stokes**

USA

**Hiroshi Taneichi**

Japan

**George H Thompson**

USA

**Marian H Wade**

USA

**M S Wong**

Hong Kong

**Fabio Zaina**

Italy

